# Temperature as a Regulator of Red Blood Cell Fate: From Membrane Dynamics to Cellular Clearance

**DOI:** 10.3390/medsci14040503

**Published:** 2026-08-20

**Authors:** Gregory Barshtein, Ivana Pajić-Lijaković, Alexander Gural

**Affiliations:** 1 Department of Biochemistry, The Faculty of Medicine, Hebrew University, Jerusalem 91120, Israel; 2 Faculty of Technology and Metallurgy, University of Belgrade, 11000 Belgrade, Serbia; iva@tmf.bg.ac.rs; 3 Blood Bank, Hadassah-Hebrew University Medical Center, Jerusalem 91120, Israel; gural@hadassah.org.il

**Keywords:** erythrocytes, hyperthermia, febrile-range temperature

## Abstract

Fever-range hyperthermia (38–41 °C) is a typical physiological response to infection, inflammation, and systemic stress. Although increased temperatures are known to affect blood rheology and erythrocyte activity, their comprehensive impact on red blood cell (RBC) structure, mechanics, and lifespan remains incompletely understood. This review summarizes current understanding of how moderate hyperthermia affects RBC membrane structure, internal behavior, mechanical properties, and clearance cues. Evidence shows that brief exposure to febrile temperatures primarily induces reversible biophysical modifications, including heightened membrane fluidity, increased membrane fluctuations, changes in hemoglobin–water interactions, and short-term improvements in deformability. These changes reflect adaptive adjustments within the membrane–cytosol–cytoskeleton system, potentially temporarily boosting microcirculatory flow. On the other hand, prolonged or repeated heat stress causes oxidative damage, hemoglobin auto-oxidation, accumulation of membrane-bound hemoglobin, band 3 clustering, cytoskeletal restructuring, calcium imbalance, and disruption of membrane lipid asymmetry. These effects weaken membrane stability and lead to vesiculation, shape changes, increased cell fragility, altered aggregation, enhanced adhesion, and activation of clearance mechanisms. A primary focus is the transition from reversible membrane softening to permanent structural damage over time. The research supports a model in which temperature affects RBC mechanics and related membrane, cytosolic, and signaling processes that influence RBC viability. We propose interpreting febrile hyperthermia as a dynamic factor that shifts RBCs from an adaptive phase to accelerated aging and removal during prolonged heat exposure. This perspective enhances our understanding of RBC behavior during fever and systemic inflammation and underscores the role of temperature in shaping erythrocyte function and lifespan.

## 1. Introduction

Red blood cells (RBCs) function within a carefully controlled environment, with normal body temperature around 37 °C. However, under conditions such as infection, inflammation, or heat stress, body temperature can rise to febrile levels of 38–41 °C. Although these temperature increases are often considered adaptive, enhancing immune responses and metabolism, their impact on erythrocyte structure and function remains poorly understood.

RBCs are highly sensitive to physical and environmental stress. Being anucleate and lacking organelles, they cannot activate transcriptional or translational responses to external signals. Instead, their responses rely on inherent biophysical characteristics and damage-response pathways. As a result, erythrocytes act as passive sensors that document environmental exposure by altering membrane mechanics, molecular arrangement, and their internal physical state. These modifications can accumulate over time, affecting both cellular function and lifespan.

Temperature is a key physical factor influencing various features of RBC structure. Even slight rises into the febrile range can affect membrane fluidity, cytoskeletal connections, ion permeability, and intracellular protein activity. Past research has explored specific RBC responses to higher temperatures, such as changes in deformability, aggregation, membrane lipid behavior, and eryptosis activation. However, these studies are mostly isolated within fields like hemorheology, membrane biophysics, cell death signaling, and transfusion science.

Consequently, there is still no comprehensive framework that explains how febrile-range hyperthermia affects RBCs’ membrane structure and function. Specifically, the connection between initial reversible biophysical responses and the later, irreversible membrane changes—such as vesiculation, modified surface composition, and increased clearance signals—has yet to be thoroughly examined.

This review consolidates current understanding of how febrile-range hyperthermia (38–41 °C) impacts RBC membrane properties. It examines the relationship between membrane mechanics, molecular organization, and intracellular interactions, emphasizing the shift from reversible physical changes to damage-induced structural remodeling. Special focus is placed on how these processes influence clearance-related phenotypes and the possible role of temperature in regulating RBC lifespan and function in physiological and pathological settings.

This review exclusively examines publications on RBC behavior in healthy individuals. Consequently, we did not include cells infected by Plasmodium falciparum. The behavior of these infected cells at febrile temperatures warrants special attention. For those interested in this subject, we recommend several relevant publications [1,2,3].

Having outlined the above considerations, it is important to distinguish between the behavior of RBCs subjected to brief exposure to 41 °C—such as during short-term incubation/measurements—and that of cells exposed to prolonged heating, whether in vivo or in vitro. These two conditions reflect fundamentally different regimes. Short-term exposure primarily reveals intrinsic, reversible biophysical responses and is therefore of particular interest from a scientific perspective. In contrast, prolonged exposure is more clinically relevant, as it captures the cumulative effects of thermal stress, including membrane remodeling and the activation of damage-related pathways. Such a delineation may not always be feasible because of the research protocols chosen by the authors.

In this review, we aim to differentiate between these two regimes and to analyze their respective contributions to RBC behavior under febrile conditions.

### 1.1. Literature Search Strategy

Next, we provide a narrative review aimed at summarizing existing knowledge on how febrile-range hyperthermia affects human RBCs, rather than conducting a systematic review in line with PRISMA guidelines.

The literature search primarily utilized PubMed, Web of Science, and Scopus, encompassing publications up to May 2026. It involved searches with various keyword combinations such as red blood cells, erythrocytes, hyperthermia, fever, febrile temperature, membrane mechanics, deformability, cytoskeleton, spectrin, band 3, hemoglobin, vesiculation, eryptosis, lipid asymmetry, aggregation, adhesion, oxidative stress, and cell clearance. Additional relevant works were found through citation tracking of key articles and review papers.

The focus was on peer-reviewed experimental studies examining how physiological febrile-range temperatures (38–41 °C) affect human RBCs. Classic studies that laid the groundwork for understanding RBC membrane mechanics, cytoskeletal structure, and hemorheology were included when they were crucial for comprehending the underlying mechanisms. Research conducted at supra-febrile temperatures (≥42 °C) was included only if it provided significant mechanistic insights and clearly indicated conditions outside the normal fever range.

This review mainly concentrates on healthy human RBCs. Studies on pathological conditions such as Plasmodium falciparum-infected erythrocytes or hereditary RBC disorders were mostly excluded, except when they provided mechanistic insights directly relevant to the fundamental membrane or cytoskeletal processes discussed.

Finally, artificial intelligence tools (ChatGPT) were used only to assist with language editing and improve readability. The identification, selection, interpretation, and critical evaluation of the scientific literature were performed exclusively by the authors.

### 1.2. Operational Definitions of Temperature and Exposure Duration

In this review, febrile-range hyperthermia is defined as 38–41 °C, matching the typical body temperatures seen during physiological fever. Temperatures of 42 °C and above are classified as supra-febrile (pathological) hyperthermia and are discussed separately when they offer mechanistic insights into how body temperature affects RBC remodeling. To clarify the timeline of thermal effects, we categorize short-term heating as exposures lasting from seconds to several minutes, during which reversible biophysical responses like increased membrane fluidity, greater membrane fluctuations, and temporary improvements in deformability dominate. Conversely, prolonged heating refers to exposures of ≥30 min, a period during which oxidative changes, membrane–cytoskeleton remodeling, hemoglobin oxidation, vesiculation, and other structural alterations are generally observed in studies [4,5,6]. The 30 min mark serves as an operational, not absolute, biological boundary—based on common experimental conditions rather than a definitive transition point. When relevant, specific studies with different exposure durations or supra-febrile temperatures are explicitly cited in the text.

### 1.3. The Key Experimental Studies Investigating the Effects of Temperature on RBCs

The literature identified through the search primarily consists of key experimental studies investigating the impact of increased temperatures on human RBCs. Due to significant variations in their experimental designs, temperature conditions, exposure durations, and analytical techniques, direct comparisons are challenging. To facilitate understanding and provide context for the subsequent sections, a summary of the main studies included in this review is presented in Table 1.

## 2. RBC State Under Febrile-Range Temperature

Febrile-range temperatures (38–41 °C) represent a common physiological and pathological condition that can significantly influence red blood cell structure and function. Although RBCs are adapted to circulate under varying mechanical and chemical stresses, even moderate thermal elevation perturbs the delicate balance between membrane, cytoskeleton, and cytosolic components. These changes affect key properties such as deformability, aggregation, membrane stability, and lifespan (see Section 3). Importantly, thermal effects are not limited to the membrane but involve integrated multiscale responses. Understanding RBC state under febrile conditions is therefore essential for interpreting microcirculatory alterations during infection, inflammation, and systemic stress.

### 2.1. Alteration of RBC Membrane and Cytosol State Under Febrile-Range Temperature: General Observation

This section examines how febrile-range temperatures influence RBC membrane mechanics, highlighting the difference between immediate adaptive responses and long-term structural damage.

*Short-term febrile heating (seconds to minutes)*. Temperature fundamentally affects membrane physical properties by altering lipid organization and protein behavior [12,13]. As temperature rises, lipid bilayers become more fluid due to increased lateral diffusion and decreased packing density, which lowers membrane viscosity and bending rigidity [7,14].

Waugh and Evans’s foundational micropipette aspiration research [7] examined how the elasticity of RBC membranes varies across a wide temperature range (2–50 °C). They assessed both the shear and area compressibility moduli, finding that membrane stiffness decreases with increasing temperature, indicating greater membrane compliance. The shear modulus exhibited a consistent negative temperature coefficient, indicating ongoing membrane softening. Additionally, thermoelastic analysis linked membrane extension to increased configurational entropy, likely influenced by the spectrin cytoskeleton. These findings offered early quantitative proof that RBC membrane mechanics are inherently sensitive to temperature [7].

These observations indicate that the membrane’s temperature-dependent elasticity is influenced not only by the lipid bilayer but also by the combined behavior of the membrane and the cytoskeletal system. In this regard, the RBC membrane functions more like a composite structure than a mere fluid bilayer, with its cytoskeletal network connected to the bilayer through anchoring complexes, including band 3 and glycophorin C, which are linked by adaptor proteins such as ankyrin and protein 4.1 [15,16]. Maintaining these vertical and horizontal interactions is essential for membrane stability, elasticity, and surface area [17,18].

Temperature elevation affects not only the lipid phase but also the strength and dynamics of protein-mediated interactions [19]. Thermal fluctuations can weaken bilayer–cytoskeleton coupling, increasing membrane undulations and reducing the effective shear modulus of the composite membrane [20,21]. At the same time, increased molecular motion may alter protein conformation and the stability of anchoring complexes [22], further affecting mechanical behavior.

*Longer-term febrile heating (tens of minutes and beyond)*. Prolonged exposure of RBCs to febrile-range temperatures induces a progressive transition from a mechanically compliant but stable state to one characterized by structural and biochemical deterioration. Unlike short-term heating, where membrane softening dominates, longer-term exposure allows time-dependent processes—particularly oxidative modifications and protein reorganization—to develop and become functionally significant. These processes fundamentally alter the coupling between the lipid bilayer and the spectrin cytoskeleton, as well as the interactions between cytosolic hemoglobin (Hb) and membrane proteins.

A central feature of longer-term febrile heating is the enhancement of Hb auto-oxidation and the subsequent formation of hemichrome-like species. Hemichromes exhibit a strong affinity for the cytoplasmic domain of band 3, leading to stable Hb–membrane association and the nucleation of band 3 clusters [23,24]. This clustering is a well-established hallmark of RBC aging and triggers downstream membrane reorganization, including phosphorylation events and altered protein–protein interactions [25]. In parallel, oxidative stress can promote additional modifications of membrane proteins and lipids, further destabilizing membrane architecture and reducing its resilience.

Sustained heating also amplifies the mechanical consequences of earlier temperature-induced softening. While elevated temperature initially reduces the membrane shear modulus [7], prolonged exposure leads to cumulative damage that impairs the membrane’s ability to withstand repeated deformation [5]. Experimental studies on mechanical fatigue demonstrate that RBCs subjected to repeated or sustained stress exhibit progressive loss of surface area, increased vesiculation, and reduced deformability [18,26]. Although these studies are not strictly thermal, they provide a mechanistic framework for understanding how a softened membrane, when maintained under stress conditions, transitions toward structural failure. In this context, febrile-range temperature can be viewed as a sensitizing factor that lowers the threshold for fatigue-induced damage.

Importantly, the accumulation of membrane-bound hemoglobin (MBHb) under prolonged heating has direct functional consequences. Elevated MBHb levels have been associated with decreased RBC deformability [27], thereby impairing microcirculatory performance. In addition, band 3 clustering and oxidative modifications act as signals for erythrocyte clearance, linking thermal stress to accelerated cellular aging and removal from circulation. Thus, longer-term febrile heating does not merely exaggerate the effects of short-term exposure but qualitatively shifts RBC behavior toward an aging-like phenotype, characterized by persistent membrane alterations, reduced mechanical stability, and increased susceptibility to clearance.

Overall, prolonged exposure to febrile-range temperatures integrates thermal, oxidative, and mechanical pathways into a unified process of RBC deterioration. This time-dependent transition highlights the importance of considering not only the magnitude of temperature but also the duration of exposure when evaluating the physiological and pathological consequences of febrile conditions on circulating erythrocytes.

*Interim conclusions*: Febrile-range temperatures have a biphasic, time-dependent impact on RBC membrane structure and mechanics, producing distinct outcomes with short-term and long-term exposures. In the initial seconds to minutes, heating primarily alters the membrane’s physical properties by increasing lipid fluidity and weakening bilayer–cytoskeleton connections. This enhances deformability and flow flexibility but reduces mechanical stability, yielding a more compliant yet vulnerable membrane.

Over longer periods—tens of minutes or more—biochemical and structural changes, such as oxidative modifications, hemichrome formation, band 3 clustering, and hemoglobin buildup on the membrane, progressively damage membrane organization and cytoskeletal integrity.

These changes convert the membrane from a softened state to a structurally impaired one with reduced deformability, increased vesiculation (for details, see Section 3.1), and heightened clearance signals. Importantly, long-term effects are not merely an extension of short-term softening but a qualitative shift toward an aging-like phenotype. Thus, fever acts as both a rapid physical modulator and a slower biochemical stressor, linking membrane mechanics, hemoglobin state inside the cells, and RBC lifespan within a unified, time-dependent framework.

In Section 2.2, Section 2.3 and Section 2.4, we explore in detail the structural, mechanical, and biochemical phenomena that occur in the RBC membrane due to heating. The focus is on how temperature-driven changes in membrane organization, cytoskeletal interactions, and hemoglobin–membrane interactions develop over time and influence RBC function.

### 2.2. Intracellular Coupling: Hemoglobin and Cytosolic Water

The interior of the RBC plays a central role in determining overall cell mechanics and should not be regarded as a passive medium [28]. RBC deformability depends not only on membrane elasticity and surface area-to-volume ratio, but also on cytoplasmic viscosity, which is strongly influenced by hemoglobin, the predominant intracellular macromolecule. Both classical and modern analysis of RBC biomechanics identify intracellular viscosity as a major determinant of deformability alongside membrane viscoelasticity and cell geometry [29]. Because Hb occupies approximately 30–35% of the cytosolic volume, the RBC interior represents a highly crowded physicochemical environment in which protein–protein and protein–water interactions become mechanically significant.

*Short-term febrile heating*: During short-term exposure to febrile temperatures, intracellular responses are dominated by rapid and largely reversible alterations in Hb dynamics and water mobility. Neutron-scattering studies of intact human RBCs have demonstrated that Hb dynamics are highly temperature-dependent in situ, indicating that moderate heating directly modifies the cell’s intracellular physical state [30]. Complementary investigations of hydrated Hb have shown that protein conformational fluctuations are strongly coupled to hydration-layer dynamics, emphasizing the importance of Hb–water interactions in regulating intracellular behavior [31].

Temperature-dependent changes also occur in cytosolic water organization. Quasi-elastic neutron-scattering experiments revealed the coexistence of hydration-layer water and bulk-like intracellular water, both exhibiting increased mobility at elevated temperature [30,31,32]. Such changes may reduce intracellular resistance and contribute to the transient increase in RBC deformability.

*Long-term febrile heating*. With prolonged or repeated exposure to febrile-range temperatures, intracellular alterations become progressively more pronounced and potentially destabilizing. Sustained thermal stress may induce persistent changes in hemoglobin organization, hydration structure, and cytoplasmic viscosity, thereby altering the mechanical coupling between the cell interior and the membrane [5,6,27,29,33,34]. This interpretation is consistent with the classical view that RBCs’ deformability depends on three interdependent parameters: cell geometry, membrane viscoelasticity, and cytoplasmic viscosity [29].

Although moderate warming initially enhances molecular mobility, prolonged temperature elevation may disrupt the balance between Hb conformational flexibility and intracellular structural stability, producing persistent impairment of RBC deformability and membrane stability [5,6,33,34,35]. Nash and Meiselman showed that heat treatment alters RBC membrane viscoelasticity and reduces deformability, indicating that thermal exposure causes lasting mechanical changes rather than only transient fluidization [6]. More recently, Matrai et al. [5] demonstrated that incubation at 40–43 °C significantly worsens RBC deformability and membrane stability, supporting the relevance of febrile-range heating to RBC mechanical impairment.

Persistent changes in hydration-layer organization and water mobility may modify intracellular energy dissipation and alter the transmission of mechanical forces from the cytosol to the membrane. As intracellular viscosity becomes increasingly heterogeneous, stress distribution within the cell may become less efficient, predisposing RBCs to fatigue and structural instability during repeated cycles in circulation.

As mentioned earlier, prolonged temperature exposure enhances Hb–membrane interactions, leading to the accumulation of membrane-bound hemoglobin and oxidative damage to the membrane [36,37]. These changes can disrupt membrane integrity, weaken connections to the cytoskeleton, and raise local mechanical stress. Evidence supporting these processes includes the finding that membrane-bound Hb decreases RBC deformability and indicates a pathological redistribution of Hb between the cytosol and the membrane [27]. In parallel, Ivanov et al. showed that membrane proteins, including spectrin and band 3, participate in temperature-induced permeability changes and thermal damage to RBCs [33].

At the same time, sustained dehydration due to temperature-related ionic imbalance may further increase intracellular Hb concentration and cytoplasmic viscosity. Ca^2+^ influx can activate Gardos channels, promoting K^+^ efflux, water loss, cell shrinkage, and an increase in intracellular Hb concentration [38,39].

Taken together, these studies support an important conceptual conclusion: temperature-induced changes in RBC mechanics arise from tightly coupled membrane–cytosol interactions, in which intracellular and membrane processes are not separate, but fundamentally interdependent [5,29,33].

### 2.3. Temperature-Dependent Hemoglobin–Membrane Interactions in Red Blood Cells

Temperature influences the interaction between hemoglobin and the RBC membrane in ways that go beyond simple effects on bulk rheology [27]. At body temperature (37 °C), Hb is primarily in the cytosol, and its association with the membrane is limited, transient, and governed by interactions with cytoskeletal proteins such as spectrin, ankyrin, and band 3 [23,37]. Under these conditions, the amount of membrane-bound hemoglobin band remains low and is largely reversible, indicating a dynamic balance regulated by redox state, cellular organization, and membrane health.

*Short-term febrile heating (seconds to minutes)*. During brief exposure to febrile temperatures, the dominant effect is a rapid alteration of membrane mechanics rather than a substantial change in Hb–membrane binding. Elevated temperature reduces membrane shear elasticity and enhances membrane fluctuations [40], reflecting partial weakening of spectrin–bilayer coupling [7,8,41]. This results in enhanced apparent deformability and a more flexible membrane state. While brief heating may slightly increase Hb auto-oxidation and transient Hb–membrane interactions, MBHb levels remain low and largely reversible, and the cell operates in a mechanically softened but not structurally compromised regime.

*Longer-term febrile heating (tens of minutes and beyond)*. In contrast, sustained exposure to febrile temperatures progressively shifts the system towards stable hemoglobin–membrane association. Prolonged heating enhances Hb auto-oxidation and promotes the formation of hemichrome-like species with high affinity for band 3 [24,42]. These interactions drive band 3 clustering, a hallmark of RBC aging and a trigger for membrane reorganization and clearance signaling [23,25]. In parallel, continued membrane softening and altered intracellular hydration further facilitate Hb–membrane interactions. As a result, MBHb accumulates and becomes functionally significant, correlating with reduced RBC deformability and impaired mechanical stability [27]. This transition reflects a shift from a reversible, compliant state to one characterized by persistent structural alterations and increased susceptibility to mechanical and oxidative stress [5].

**Figure 1:**
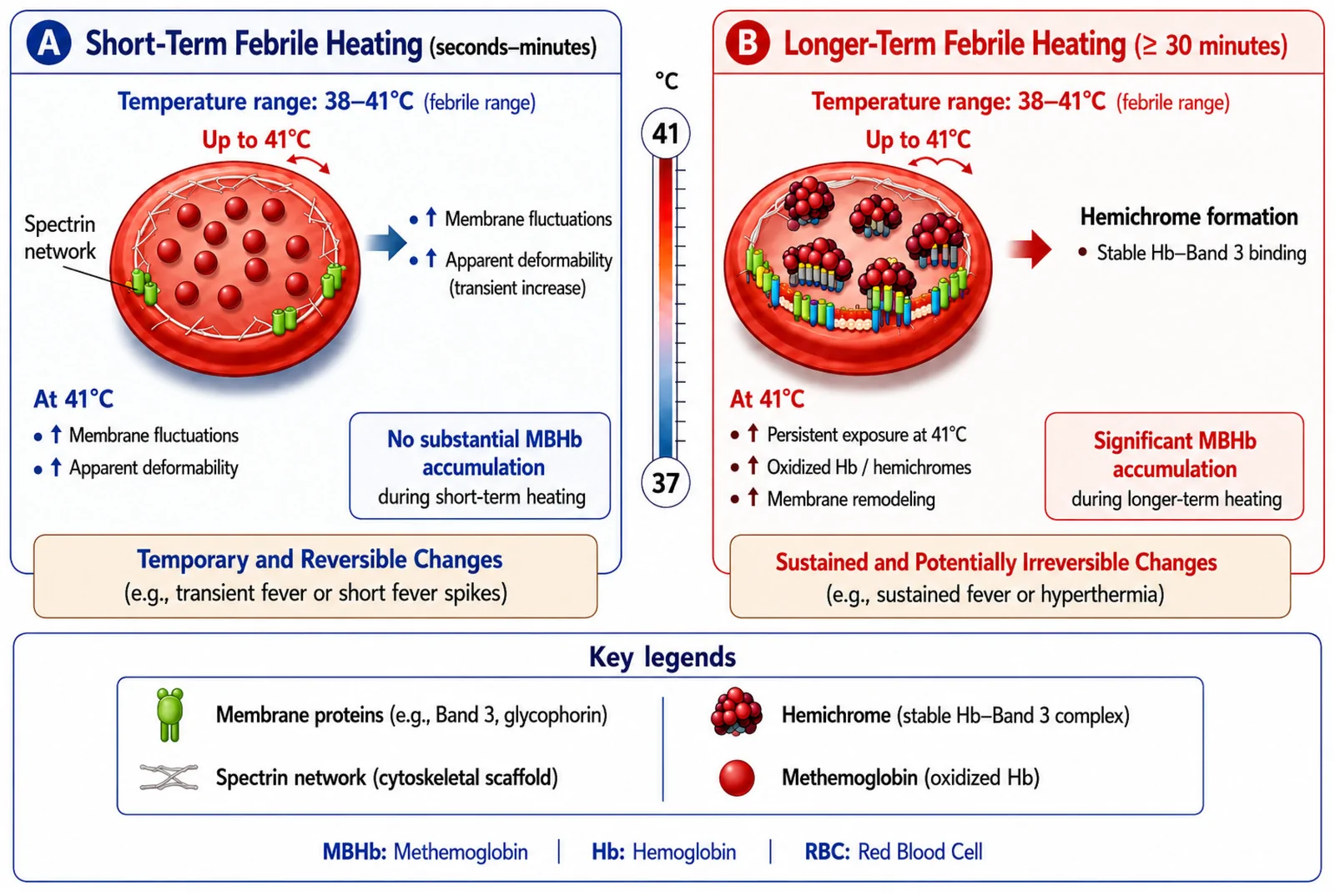
Time-dependent effects of febrile temperature on hemoglobin–membrane interactions in red blood cells (RBCs). This schematic depicts how short- and longer-term febrile heating (38–41 °C) affect hemoglobin (Hb) arrangement and membrane structure in RBCs differently. (**A**) Short-term febrile heating (seconds to minutes): Brief exposure primarily affects membrane mechanics by decreasing shear elasticity and increasing fluctuations, likely due to a temporary weakening of spectrin–bilayer interactions. These modifications enhance deformability without significant structural damage. Hemoglobin mostly remains soluble in the cytosol, and membrane-bound hemoglobin (MBHb) levels stay low and reversible, indicating a mechanically softened yet intact membrane. (**B**) Longer-term febrile heating (≥30 min): Prolonged exposure leads to hemoglobin oxidation and formation of hemichrome-like species with high band 3 affinity. Hemichromes binding to band 3 cause protein clustering, membrane domain reorganization, and MBHb accumulation. Red spheres depict cytoplasmic hemoglobin molecules. Colored channels show band 3-containing complexes that anchor the membrane to the spectrin cytoskeleton. Extended heating promotes hemichrome–band 3 interactions, driving membrane remodeling and senescence-related changes that may impair erythrocyte function. Figure created by the authors using an AI-assisted graphic design tool [ChatGPT, version 5.2].

Simultaneous membrane softening and changes in intracellular conditions further promote Hb–membrane associations. This leads to reduced cell deformability, increased fragility, and a higher likelihood of removal from circulation, resembling an aging RBC phenotype (see Figure 1).

### 2.4. Calcium Influx and Ion Imbalance

Elevated temperatures can disrupt the ionic balance in RBCs by increasing membrane permeability and activating temperature-sensitive, mechanosensitive, or stress-responsive ion channels. Although mature RBCs lack nuclei and mitochondria, they have tightly regulated ion transport systems that are vital for maintaining cell volume, membrane asymmetry, deformability, and lifespan. Notably, Ca^2+^ entry is not just a pathological event: Hudec et al. [43] showed that human RBCs display a baseline Ca^2+^ influx under normal conditions, suggesting that the erythrocyte membrane is constantly exposed to low-level Ca^2+^ entry, which must be offset by active extrusion. During fever-like or hyperthermia states, even a small increase in this basal Ca^2+^ permeability can have significant effects, since RBCs normally keep intracellular Ca^2+^ levels very low. Therefore, thermal stress can shift the balance from normal Ca^2+^ regulation towards overload, triggering processes such as phospholipid scrambling, K^+^ efflux through Gardos channels, cellular dehydration, membrane remodeling, and vesiculation. Consequently, Ca^2+^ influx serves as a crucial trigger, converting temperature-induced membrane changes into structural and functional alterations in RBCs [38,39,43,44].

One important pathway involves activation of non-selective cation conductance, including mechanosensitive channels such as Piezo1 and other stress-sensitive permeability pathways. Such channels can be activated by membrane deformation, oxidative stress, or altered membrane tension, all of which may occur during heating and fever-associated circulatory stress. Increased Ca^2+^ entry initiates several downstream events that resemble eryptosis, the programmed death-like response of erythrocytes [38]. In particular, Ca^2+^ activates scramblase and inhibits aminophospholipid translocase activity, resulting in phosphatidylserine (PS) exposure on the outer leaflet of the RBC membrane. PS-positive RBCs are more adhesive, more readily recognizable by macrophages, and may contribute to procoagulant activity [45,46].

Ca^2+^ influx also activates the Gardos channel, a Ca^2+^-dependent K^+^ channel. Opening of the Gardos channel causes K^+^ efflux, followed by Cl^−^ and water loss, leading to RBC dehydration and shrinkage. This volume reduction increases mean cellular hemoglobin concentration, raises cytoplasmic viscosity, and impairs deformability. These changes are particularly relevant under fever-range temperatures, where cytosolic hemoglobin organization and water dynamics may already be altered. The combined effects of dehydration, increased intracellular viscosity, and membrane remodeling can therefore compromise RBC passage through capillaries and splenic slits.

In parallel, elevated Ca^2+^ may activate calpain, promoting proteolytic remodeling of cytoskeletal proteins and weakening the membrane–cytoskeleton interface. This contributes to echinocyte formation, vesiculation, and progressive loss of membrane surface area. These processes are consistent with studies showing that thermal stress promotes RBC shape transformation, vesicle shedding, and reduced mechanical stability [5,6,11]. Overall, calcium influx and ionic imbalance provide a mechanistic link between febrile-range heating, membrane instability, vesiculation, dehydration, and impaired RBC deformability.

### 2.5. RBC Membrane Lipid Asymmetry Under Fever-Range Temperature

**Figure 2:**
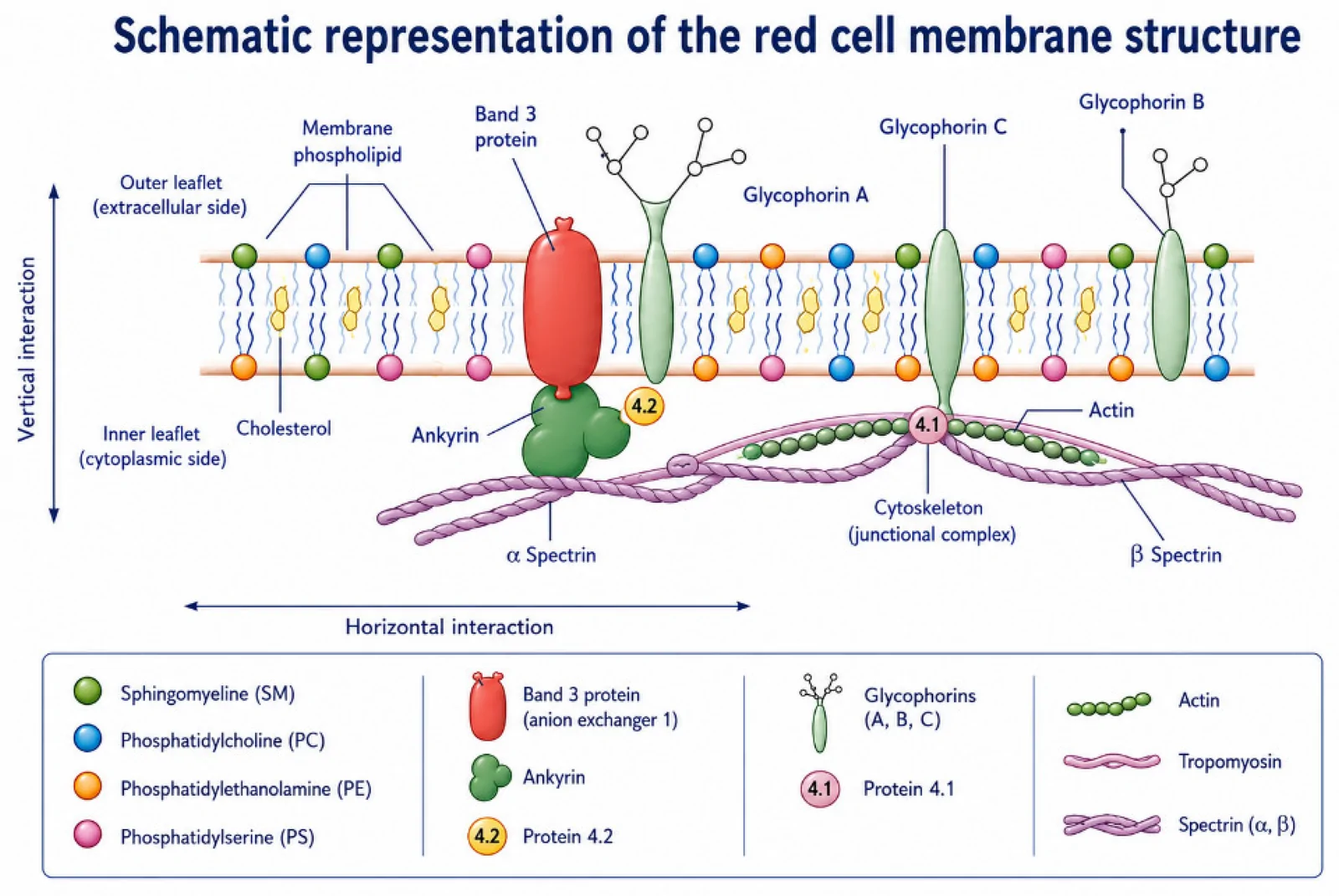
Structure of the red blood cell membrane and its lipid asymmetry. The RBC membrane comprises a lipid bilayer attached to a spectrin-based cytoskeleton beneath. The bilayer exhibits asymmetry, with phosphatidylcholine (PC) and sphingomyelin (SM) primarily on the outer (extracellular) surface, while phosphatidylserine (PS) and phosphatidylethanolamine (PE) are mostly on the inner (cytoplasmic) side. Cholesterol is present in both leaflets, helping maintain fluidity and stability. Integral proteins such as band 3 (anion exchanger 1) and glycophorins (A, B, C) span the membrane and connect to the cytoskeleton via adaptor proteins such as ankyrin and protein 4.1/4.2, which link the membrane to the spectrin–actin network. This cytoskeleton, composed of α- and β-spectrin, actin, and related proteins such as tropomyosin, provides mechanical strength, flexibility, and durability during deformation. Both vertical interactions (between the bilayer and the cytoskeleton) and horizontal interactions (within the cytoskeletal network) help preserve membrane integrity, surface area, and flexibility. Maintaining lipid asymmetry, especially keeping PS inside the inner leaflet, is crucial for preventing inappropriate cell adhesion and ensuring proper RBC circulation. Figure created by the authors using an AI-assisted graphic design tool [ChatGPT, version 5.2].

Membrane lipid asymmetry is a key structural and functional feature of the RBC (see Figure 2), maintained under physiological conditions by ATP-dependent aminophospholipid translocase activity and tightly regulated interactions between membrane proteins and the lipid bilayer [16,47]. In healthy RBCs, phosphatidylserine is predominantly confined to the inner leaflet, and its externalization is minimal in the absence of stress or eryptotic signaling [48]. This asymmetric organization is essential for maintaining membrane stability, deformability, and non-adhesive behavior in circulation [47,49,50].

*Short-term febrile heating (seconds or minutes)*. As indicated by research, within the narrow fever range of 38–41 °C, temperature alone does not immediately impair membrane asymmetry in healthy RBCs. Instead, brief febrile episodes mainly affect membrane mechanics rather than lipid topology. The strongest evidence for temperature-dependent loss of asymmetry comes from cases in which febrile exposure is combined with other stressors or with sufficient incubation time (see below). These observations suggest a threshold-like, time-dependent process rather than immediate scrambling from 38 to 41 °C being operative in healthy cells [8,51]. Mechanistically, febrile-range temperatures act as sensitizing factors, and while they may by themselves weaken the stability of asymmetry control, significant PS externalization probably needs additional factors such as oxidative stress, Ca^2+^ overload, and ATP depletion [52,53,54], leading to progressive loss of asymmetry and an eryptosis-like phenotype [48,55].

*Longer-term febrile heating (tens of minutes and beyond)*. In contrast to short-term exposure, longer incubation at fever-range temperatures can progressively destabilize lipid asymmetry through time-dependent mechanisms. Available evidence indicates that sustained exposure lowers the threshold for loss of asymmetry. For example, while no detectable increase in PS exposure was observed in normal RBCs after 2 h at 40 °C, earlier studies have shown that prolonged incubation (on the order of 24 h) at similar temperature leads to a measurable increase in surface PS [51]. These observations indicate that the effect of febrile exposure on lipid asymmetry is governed by both temperature range and duration.

Longer-term heating is likely to impair the maintenance of membrane asymmetry through several converging pathways. Gradual ATP depletion may reduce flippase activity, while increased oxidative stress and alterations in intracellular Ca^2+^ homeostasis can activate lipid scrambling pathways [56]. Experimental and mechanistic studies indicate that PS externalization requires both mechanisms [54,57]. In parallel, temperature-induced changes in membrane protein conformation and bilayer–cytoskeleton interactions may further destabilize lipid organization [4]. Functionally, loss of lipid asymmetry contributes to increased cell adhesion, recognition by macrophages, and enhanced clearance from circulation, consistent with eryptotic signaling pathways [56]. Thus, in the febrile-range temperature window, prolonged exposure acts not as an immediate trigger but as a facilitator of the breakdown of asymmetry, integrating thermal, metabolic, and oxidative effects into a time-dependent transition toward RBC aging and functional decline.

*Interim conclusions*: Within the febrile-range temperature window (38–41 °C), membrane lipid asymmetry in red blood cells remains preserved during short-term exposure and is not directly disrupted by temperature alone. Instead, febrile heating primarily affects membrane mechanics, while lipid topology remains largely intact for short durations. The loss of asymmetry is a time-dependent, multifactorial process that requires sustained exposure and can be exacerbated by additional stressors, including oxidative imbalance, Ca^2+^ influx, or ATP depletion. Extended heating lowers the threshold for disrupting asymmetry by impairing flippase activity, encouraging lipid scrambling, and destabilizing membrane–cytoskeleton interactions. This results in the gradual externalization of phosphatidylserine and an eryptosis-like phenotype. Therefore, febrile-range temperature acts not as a direct cause but as a sensitizer, integrating thermal, metabolic, and oxidative factors into a gradual progression toward RBC aging, functional decline, and removal.

### 2.6. Temperature-Dependent Remodeling of RBC Mechanics and Spectrin Architecture

Our review of existing literature suggests that cytoskeletal restructuring occurs over relatively extended periods, typically tens of minutes (*Longer-term heating*).

Two studies provide complementary evidence that febrile-range temperatures influence RBC mechanics by altering membrane–cytoskeleton structure. Kozlova et al. [4] visualized the spectrin matrix with atomic force microscopy (AFM) after 30 min of incubation at varying temperatures. At 36–37 °C, RBC morphology and spectrin structure stayed close to the normal network, with spectrin elements around ~150 ± 60 nm. However, at 39–40 °C, RBC shape shifted, with echinocytes and target cells starting to appear, and the spectrin network divided into regular and irregular patterns. Network elements grew to ~220–240 nm, and nanodefects appeared at actin and ankyrin junctions. At 42–43 °C, irregular spectrin patterns dominated, affecting about 83% of the matrix, indicating significant cytoskeletal reorganization.

Sheikhhassani et al. [9] extended these structural insights to function by examining single-cell mechanics with optical tweezers. The authors found that RBC stiffness depends on temperature and strain rate: cells were stiffest at room temperature and softer at febrile temperatures, with 40 °C reducing stiffness across different stretching speeds. Notably, atorvastatin lowered RBC stiffness at 37 °C but not at 40 °C, suggesting that febrile temperatures may already maximize softening. The study links this loss of drug effect at higher temperatures to cytoskeletal disruptions, including defective actin, ankyrin gaps, and a disordered spectrin network. Overall, these findings support the idea that febrile-like heating induces a softening–destabilization shift: RBCs become more flexible, but this reflects ongoing disruption of spectrin-based membrane organization rather than merely improved deformability.

**Conclusion to Section 2: Temperature as a regulator of coupled membrane–cytosol–cytoskeleton dynamics.**

**Figure 3:**
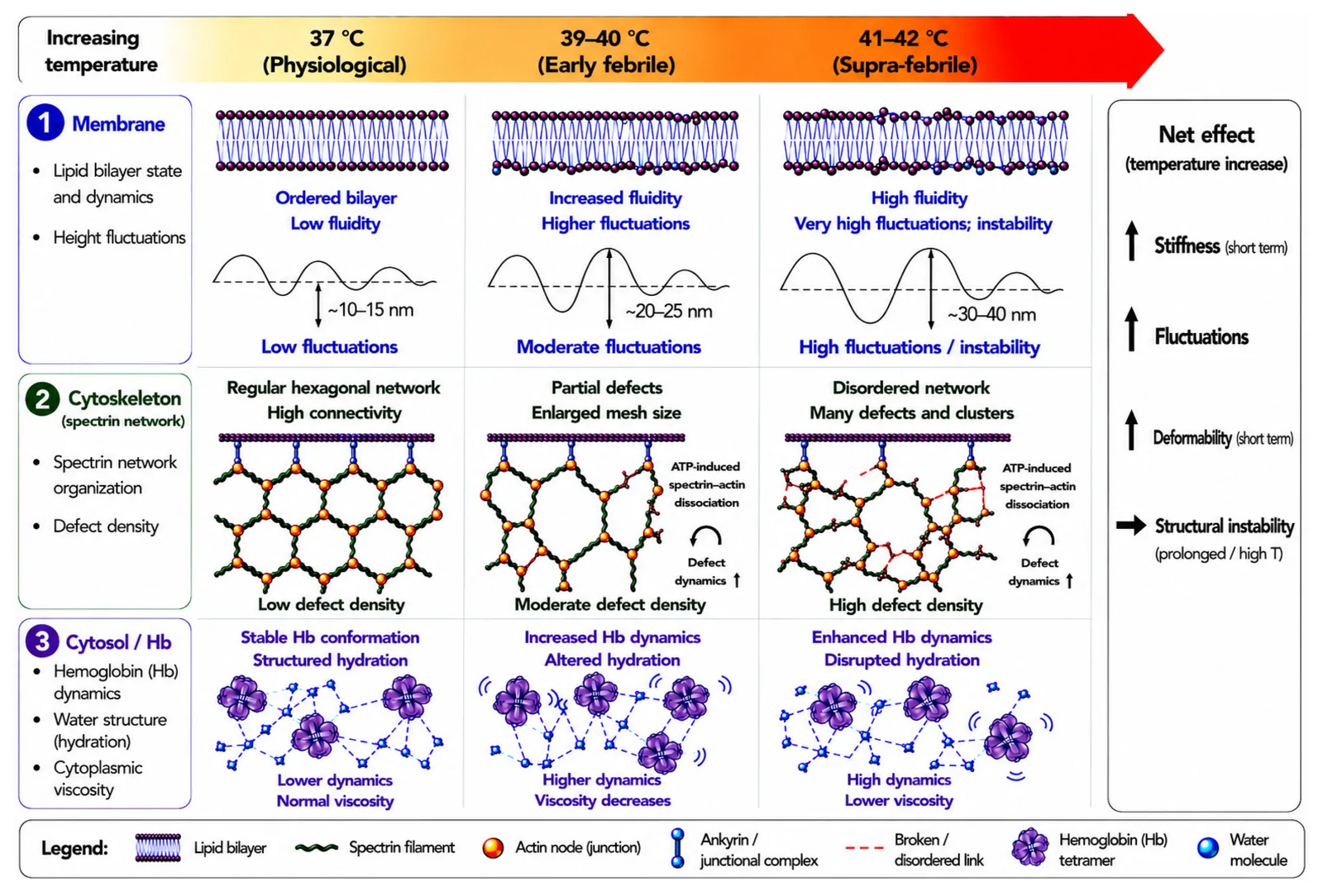
Temperature-driven coupling between membrane structure, cytoskeletal remodeling, and intracellular dynamics in red blood cells. Schematic overview illustrating how increasing temperature coordinately influences the three principal structural compartments of the red blood cell (RBC): the membrane, the spectrin-based cytoskeleton, and the intracellular hemoglobin–water environment. Under physiological conditions (37 °C), the membrane exhibits relatively low fluidity and limited fluctuations, the spectrin network remains highly ordered with low defect density, and hemoglobin is associated with a structured hydration shell, supporting normal cytoplasmic viscosity and stable mechanical behavior [7,30,31,32]. During early febrile temperatures (39–40 °C), membrane fluidity and fluctuations increase [7,40]; ATP-dependent spectrin–actin dissociation promotes partial cytoskeletal reorganization and enlargement of the spectrin mesh [4,9]; and enhanced hemoglobin dynamics together with altered hydration modify intracellular viscosity and mechanical coupling [30,31,32]. At higher temperatures (41–42 °C), these processes become more pronounced, resulting in extensive membrane fluctuations, disruption of the spectrin network with increased defect density and clustering [4], and marked changes in hemoglobin hydration and intracellular dynamics [30,31,32]. The right-hand panel summarizes the functional coupling between membrane fluidity, cytoskeletal remodeling, and intracellular physicochemical changes, highlighting how their coordinated interaction determines the overall mechanical response of RBCs to thermal stress [5,7,29]. Collectively, these temperature-dependent processes reduce membrane stiffness, transiently enhance deformability during short-term heating, and, following prolonged or more intense thermal exposure, promote structural instability and mechanical deterioration [5,6,11]. This figure is a conceptual synthesis of experimentally established mechanisms reported in the cited literature and summarizes qualitative relationships only; it does not present original quantitative measurements or experimental datasets. Figure created by the authors using an AI-assisted graphic design tool [ChatGPT, version 5.2].

Febrile-range temperatures influence RBC behavior by altering the membrane, cytoskeleton, and internal environment (Figure 3). Elevating the temperature to approximately 40–41 °C increases membrane fluctuations and reduces stiffness, but prolonged exposure can cause structural instability. AFM results show a gradual disorganization of the spectrin network, with larger mesh sizes and the formation of nanodefects at actin–ankyrin junctions. Single-cell tests reveal a corresponding reduction in mechanical stiffness. These structural changes may be linked to ATP-driven cytoskeletal dynamics, in which brief spectrin-actin dissociations create local defects, weaken connections, and allow membrane movement. Temperature likely amplifies these effects by promoting defect formation and reorganization, yielding a more compliant yet less stable cytoskeleton. At the same time, temperature affects the cell’s internal environment, including Hb conformations and water structure, which alter cytoplasmic viscosity and force transmission. These changes increase membrane fluctuations and deformability. Overall, the softer RBCs at higher temperatures reflect both membrane fluidization and reorganization within the cell and its cytoskeleton. Evidence supports a model in which temperature acts as a key regulator of interactions among the membrane, cytosol, and cytoskeleton, shifting the cell from a stable, cohesive state to a more dynamic but fragile condition during febrile episodes.

## 3. Consequences of Heating-Related RBC Remodeling on Their Mechanical Properties

### 3.1. RBC Vesiculation Under Febrile-Range Temperature

The production of extracellular vesicles is a well-known aspect of RBC function [58]. During their lifespan, erythrocytes continuously release small membrane vesicles, resulting in the loss of part of their membrane and internal components [59,60]. This process is widely regarded as a crucial means of maintaining cellular integrity by eliminating damaged proteins, oxidized molecules, and other potentially harmful substances [60,61]. Wagner et al. [62] later described vesiculation as a typical physiological event occurring in an RBC membrane. Greenwalt [35] focused on vesicle shedding as a mechanism by which RBCs eliminate damaged or destabilized membrane components.

Broadly, temperatures around 38–41 °C accelerate membrane remodeling processes that naturally occur during RBC aging and storage. Vesiculation serves as a protective mechanism by removing damaged components, including oxidized hemoglobin, altered membrane proteins, and senescence-related band 3 epitopes. This process delays hemolysis but causes continuous membrane loss and reduced cell flexibility.

Febrile-range temperatures can directly promote RBC vesiculation by changing the thermodynamic state of the hydrated membrane.

Vodyanoy [10] showed that vesicle shedding at higher temperatures is not merely a passive consequence of membrane damage but a thermodynamically driven process linked to erythrocyte shape changes and the entropy–enthalpy balance. Earlier microscopic studies by the same team have also indicated that vesicle concentration roughly triples as temperature increases from 38 to 40 °C, directly linking febrile-range heating to increased vesicle release. These results underscore the importance of the hydrated physical state of the RBC membrane as a critical factor in heat-induced vesiculation and as a sensitive marker of erythrocyte stability under thermal stress [10].

An independent study by Moore et al. [11] provided direct experimental evidence for this hypothesis by using high-resolution light microscopy to observe live, unfixed blood and visualize how RBCs change in response to temperature in real time. When the core temperature increased from 38 to 40 °C, there was a significant rise in both echinocyte formation and free vesicle concentration, with vesicle numbers tripling. Vesicle release was closely linked to the discocyte–echinocyte transition, suggesting that thermal stress disrupts membrane stability and encourages budding from spiculated cells. Importantly, the authors noted that vesiculation only plays a small role in the overall loss of erythrocyte surface area during aging. This points to other mechanisms, possibly involving leukocytes, which help remove damaged membrane material.

Multiple additional studies have directly connected increased temperatures with membrane damage and vesicle formation. Araki et al. [63] demonstrated that, depending on temperature, vesiculation can occur under hypertonic conditions, highlighting lipid segregation as a key process.

It becomes particularly evident during prolonged heating, as higher temperatures disrupt the balance among the lipid bilayer, spectrin cytoskeleton, and cytosolic hemoglobin. Increased membrane fluidity and protein mobility facilitate local bending and vesicle formation. Moreover, reorganizing or weakening interactions between the bilayer and cytoskeleton decreases the energy required for vesicle formation.

At the molecular level, vesiculation is closely linked to hemoglobin oxidation and alterations in band 3 proteins. Oxidative stress induces phosphorylation and clustering of band 3, which can lead to partial detachment from cytoskeletal anchors, creating membrane regions prone to budding. Research shows that changes in band 3 and exposure of PS are crucial for microvesicle formation [64]. Additionally, an increase in intracellular Ca^2+^—caused by increased membrane permeability or activation of cation channels—stimulates scramblase activity and inhibits flippase activity, leading to PS externalization. PS-positive RBCs and vesicles are recognized by macrophages and exhibit procoagulant properties, indicating that vesiculation during fever may affect systemic functions beyond causing mechanical damage [45].

Vesiculation serves as both a beneficial and a detrimental response. Moderate vesicle release helps maintain cell integrity by eliminating damaged components, whereas excessive vesiculation reduces membrane surface area, makes the cell more spherical, and decreases deformability. This can hinder passage through capillaries and splenic filtration. Multi-omics analyses of stored RBCs reveal that vesiculation involves coordinated alterations in protein structure, membrane organization, lipid distribution, and metabolism [65]. Thus, vesiculation of RBCs at febrile-range temperatures is a comprehensive thermal-stress response that links hemoglobin instability, band 3 clustering, PS exposure, membrane budding, and the gradual decline in mechanical resilience.

### 3.2. Alteration of RBC Morphology Under Febrile-Range Temperature

One of the results of RBC vesiculation initiated by febrile-range RBC heating is alteration of cell shape. Under physiological conditions, RBCs maintain a biconcave discocyte shape that optimizes deformability and surface-to-volume ratio. However, even moderate increases in temperature can destabilize this equilibrium, promoting transitions toward echinocytic and, in more advanced stages, spherocytic forms [66,67].

Experimental observations consistently show that heating to 39–41 °C increases the fraction of echinocytes—cells characterized by spiculated membranes with regularly distributed protrusions. As previously mentioned, Moore et al. have demonstrated that elevating core temperature from 38 to 40 °C markedly increases echinocyte formation, accompanied by enhanced vesicle shedding, indicating a direct link between thermal stress and membrane remodeling [11]. This discocyte–echinocyte transition is initially reversible but becomes progressively irreversible with prolonged exposure [68].

The underlying mechanisms involve both membrane and cytoskeletal components (Figure 3). Elevated temperature increases lipid bilayer fluidity and lateral mobility of membrane proteins, while weakening vertical interactions between band 3, ankyrin, and the spectrin network [15,69]. Such changes facilitate local membrane curvature and spicule formation. In parallel, alterations in membrane potential and its temperature dependence have also been implicated in RBC shape transformations [70].

Importantly, RBC shape changes under febrile conditions cannot be attributed solely to membrane effects. Temperature-dependent alterations in cytosolic properties—particularly hemoglobin organization, hydration, and viscosity—play a critical role. Heating modifies hemoglobin conformational states and cytoplasmic water dynamics, thereby affecting intracellular viscosity and the transmission of mechanical forces to the membrane [5,33]. These effects contribute to decreased mechanical stability and increased susceptibility to shape transitions.

Calcium homeostasis provides an additional regulatory layer. Elevated temperatures can increase membrane permeability or activate cation channels, leading to intracellular Ca^2+^ accumulation. This activates scramblase and calpain, promoting phosphatidylserine exposure and cytoskeletal remodeling, thereby further destabilizing membrane structure and favoring echinocyte formation [38,46].

Functionally, these morphological transitions represent a trade-off between adaptability and stability. However, this trade-off is time-limited: while mild temperature-induced changes may transiently enhance membrane fluctuations and adaptation, longer-term febrile heating leads to reduced surface area, increased sphericity, and impaired deformability [5,6]. This compromises microcirculatory flow and splenic passage, particularly under pathological conditions such as infection or systemic inflammation.

Overall, RBC shape at febrile-range temperatures reflects an integrated, multiscale response in which membrane fluidity, cytoskeletal coupling, cytosolic viscosity, and ionic regulation collectively determine the balance between reversible adaptation and irreversible structural damage.

## 4. Functional Consequences: Mechanics, Aggregation, and Adhesion

The structural changes described above translate into complex functional outcomes.

- Deformability: Initially enhanced due to increased fluidity but reduced with prolonged exposure as structural damage accumulates.
- Fragility: Translated into increased susceptibility to mechanical stress and hemolysis.
- Aggregation: May be increased under specific conditions.
- Adhesion: Enhanced interactions with endothelial cells and plasma proteins.

These effects often conflict. Febrile-range temperatures can temporarily enhance microcirculatory flow but may also create conditions that hinder flow and elevate vascular interactions.

### 4.1. Alteration of RBC Deformability Under Febrile-Range Heating

*Short-term febrile heating*: Experimental studies consistently show that moderate warming enhances RBC deformability [71]. At first glance, this effect appears beneficial for microcirculatory flow because more deformable RBCs can traverse narrow capillaries more easily. However, this interpretation is incomplete, since the increased deformability reflects a shift toward a more compliant membrane state operating closer to the threshold of mechanical stability.

Direct measurements of membrane mechanics affirm this perspective. Waugh and Evans [7] observed a 20% reduction in the membrane shear elastic modulus between 23 °C and 41 °C, indicating notable softening of the membrane. Likewise, Park et al. [8] found decreased membrane stiffness at 41 °C compared to 37 °C, along with a roughly 50% increase in membrane fluctuations. Notably, the increase in fluctuations exceeds what thermal energy (kBT) alone would predict [72,73], indicating that structural reorganization of the membrane–cytoskeleton complex [74], rather than mere thermal motion, drives this response. These results suggest a possible temperature-dependent reorganization of spectrin and membrane–cytoskeleton interactions [75,76].

Importantly, the effects of temperature are not uniform across all modes of deformation. While shear deformability may improve, resistance to other forms of stress—including stretching, compression, and transient flow-induced loading—may already be declining [18,77]. Foo et al. [78], using laser-tweezer techniques, showed that RBC deformability between 37 °C and 42 °C particularly increases at elevated flow velocities, emphasizing the importance of thermal–hydrodynamic coupling.

At the cellular level, these early mechanical changes are closely linked to temperature-dependent modifications in cytosolic properties. Elevated temperature alters hemoglobin conformation, intracellular viscosity, and water dynamics, thereby influencing force transmission between the cytosol and the membrane. Consequently, the apparent increase in deformability likely reflects not only membrane softening but also reduced internal resistance to deformation. Mild increases in Ca^2+^ permeability and early cytoskeletal remodeling may further contribute to this transiently compliant state.

Thus, short-term febrile heating promotes a mechanically adaptive phenotype characterized by increased compliance and improved flow adaptability. However, this enhanced flexibility simultaneously indicates a reduction in the mechanical safety margin of the RBC membrane.

*Longer-term febrile heating*: With prolonged or repeated exposure to febrile-range temperatures, the initially adaptive increase in deformability progressively transitions into mechanical instability. Sustained membrane softening, persistent cytoskeletal remodeling, and cumulative intracellular alterations reduce the structural resilience of RBCs and impair their ability to tolerate repeated mechanical loading (see Section 2).

Although elevated temperature initially lowers membrane stiffness, prolonged exposure weakens the coordinated mechanical coupling between the lipid bilayer and spectrin network. Under these conditions, the membrane becomes increasingly susceptible to fatigue-related damage, vesiculation, and irreversible structural remodeling. Puig-de-Morales-Marinkovic et al. [79] demonstrated that increased membrane compliance can be accompanied by reduced structural robustness under sustained mechanical stress, supporting the concept that thermally softened RBCs become more vulnerable during repetitive circulation through the microvasculature.

Longer-term heating also amplifies anisotropic mechanical dysfunction. While cells may retain relatively high shear deformability, their resistance to extensional, compressive, or cyclic mechanical stresses declines further [18,77]. This behavior reflects the composite nature of the RBC membrane, in which different structural elements contribute differently depending on the mode and duration of loading [74,80,81].

Persistent cytosolic changes contribute further to long-term instability. Temperature-dependent alterations in hemoglobin organization, intracellular hydration, and cytoplasmic viscosity progressively modify intracellular force transmission and increase membrane stress. Elevated intracellular Ca^2+^ and sustained activation of signaling pathways involved in cytoskeletal remodeling further destabilize the membrane architecture. Over time, these processes promote vesiculation, loss of membrane surface, and irreversible remodeling.

Here, again, exposure to febrile-range temperature induces a fundamental mechanical trade-off [7,8,74]. Short-term heating enhances compliance and microvascular adaptability, but prolonged exposure reduces mechanical resilience and predisposes RBCs to membrane damage, vesiculation, and premature clearance [82]. In this context, increased deformability should be interpreted not solely as a beneficial adaptation but also as an early indicator of thermally induced mechanical vulnerability.

### 4.2. RBC Fragility Under Febrile-Range Temperature

*Short-term febrile heating* involves brief exposure to higher temperatures (≈38–41 °C), causing RBCs to undergo rapid, initially reversible physicochemical changes. Early research showed that thermal stress raises osmotic and mechanical fragility and leads to more spherical cell shapes [83]. In this context, fragility refers to the susceptibility of RBCs to rupture under osmotic, mechanical, or oxidative stress. At the membrane level, moderate heating increases lipid bilayer fluidity and boosts the lateral mobility of membrane proteins. These changes temporarily alter membrane viscoelasticity and may briefly mitigate membrane deformation while simultaneously inducing mild echinocytic transformation and controlled vesiculation. Moderate vesicle shedding may initially play a protective role by removing damaged membrane regions and oxidized components, thereby delaying catastrophic membrane failure [62,84]. Cytosolic changes also emerge rapidly. Elevated temperature alters hemoglobin conformation, intracellular hydration, and cytoplasmic viscosity, thereby modifying how mechanical forces are transmitted through the cell interior [32,85]. Under transient heating conditions, these alterations are generally modest and may partially reverse after restoration of normal temperature.

*Under longer-term febrile heating* with prolonged or repeated exposure to fever-range temperatures, RBC damage becomes progressively cumulative and less reversible. Baar [86] and Loebl et al. [87] demonstrated that sublethal thermal injury can produce delayed erythrocyte destruction, indicating persistent membrane damage even after heating ceases. Gershfeld and Murayama [35] later showed that RBC membrane bilayers become increasingly temperature-sensitive at elevated temperatures, predisposing cells to hemolysis.

Sustained heating disrupts both lipid organization and cytoskeletal integrity. Prolonged elevation of membrane fluidity weakens vertical interactions between the lipid bilayer and the spectrin-based membrane skeleton, reducing resistance to mechanical stress. Nash and Meiselman [6] reported that heat-treated RBCs become mechanically rigid and less deformable despite increased lipid mobility, indicating deterioration of global membrane viscoelastic properties. Similarly, Ivanov et al. [33] proposed that thermal alterations in membrane protein complexes contribute directly to increased fragility and destabilization.

Longer-term heating also amplifies cytosolic contributions to fragility, including chronic Ca^2+^ influx and sustained Gardos channel activation, with progressive K^+^ loss, dehydration, elevated mean corpuscular hemoglobin concentration (MCHC), and increased intracellular viscosity [88,89]. These processes stiffen RBCs further and markedly increase susceptibility to mechanical trauma and hemolysis [90].

Vesiculation also assumes a dual, ultimately detrimental role during longer-term febrile heating. While initial vesicle shedding may delay damage progression, excessive membrane loss progressively reduces surface area and increases cell sphericity, thereby elevating osmotic fragility and impairing passage through narrow capillaries and splenic slits. Recurrent febrile episodes may therefore gradually compromise RBC membrane integrity and shorten cell lifespan.

Overall, RBC fragility at febrile-range temperatures results from a multiscale process that includes membrane destabilization, cytoskeletal weakening, ionic imbalance, vesiculation, and cytosolic remodeling. All of these possess significant implications for microcirculatory flow, oxygen delivery, and early cell removal during fever and inflammation.

### 4.3. Alteration of RBC Aggregation Under Fever-Range Heating

RBC aggregation plays a key role in determining blood viscosity at low shear rates and in influencing microvascular flow [91]. This process is influenced by plasma components, primarily fibrinogen and other high-molecular-weight proteins, as well as the inherent properties of cells, such as membrane charge, deformability, shape, and surface organization [91,92,93,94]. Since these factors are temperature-dependent, fever-range heating can modify RBC aggregation.

*Short-term febrile heating*: During brief exposure to febrile temperatures, changes in aggregation mainly result from reversible physicochemical effects. Elevated membrane fluidity and lipid mobility temporarily disrupt ordered membrane domains and affect protein distribution, leading to slight modifications in cell–cell interaction forces. Simultaneously, temperature-dependent shifts in plasma protein conformation and adsorption kinetics—especially fibrinogen—may impact bridging interactions between cells [92,95].

Lim et al. [96] conducted a comprehensive rheological study to understand how temperature affects RBC aggregation and blood viscosity. They found that the apparent changes in aggregation indices as temperature decreases are partly attributable to the simultaneous increase in plasma viscosity. Once aggregation parameters were adjusted for plasma viscosity, the relationship between temperature and aggregation appeared less consistent. Nonetheless, both the threshold shear rate and shear stress required to break up RBC aggregates rose as temperature decreased, suggesting that RBC aggregates become more resistant to hydrodynamic forces at lower temperatures [96,97].

Simultaneously, early morphological changes, including echinocytic transformation and mild vesiculation, may reduce the effective contact area between neighboring RBCs, thereby weakening rouleaux stability [10,11]. Because discocyte–echinocyte transitions can be reversible under certain conditions, brief febrile heating is likely to induce mainly subtle and transient changes in RBC aggregation rather than persistent remodeling [98,99].

*Longer-term febrile heating*: Prolonged exposure to fever-like temperatures enhances RBC aggregation due to structural and biochemical changes. Continuous thermal stress induces vesiculation, irreversible shape shifts such as from echinocyte to spherocyte, and modifications in membrane protein arrangement, including band 3 clustering and cytoskeletal weakening [5,11]. These alterations can greatly affect membrane surface characteristics, including charge distribution and receptor accessibility.

Furthermore, longer-term febrile heating might accelerate oxidative reactions and promote phosphatidylserine exposure, which can alter cell interactions and potentially lead to abnormal or permanent aggregation. Changes in cell deformability are also important: stiffer cells struggle to form and break down rouleaux efficiently, resulting in different aggregate structures and formation rates [29].

Functionally, these cumulative effects are especially important in low-shear microcirculatory environments, where changes in aggregation can raise flow resistance, disturb local hematocrit distribution, and hinder oxygen delivery [99,100]. Therefore, while short-term febrile heating primarily causes reversible changes in aggregation, prolonged exposure leads to more lasting, and possibly pathological, alterations in RBC aggregability and microvascular flow properties.

### 4.4. Alteration of RBC Adhesion Under Febrile-Range Heating

Red blood cell adhesion to endothelial cells and plasma components is normally minimal under physiological conditions and is tightly regulated by membrane organization, surface charge, glycocalyx integrity, deformability, and the asymmetric distribution of membrane phospholipids [16,101,102,103,104]. Under pathological conditions, however, RBCs may acquire adhesive properties that contribute to impaired microcirculatory flow, endothelial activation, thrombosis, and inflammation [16,49]. Febrile-range temperatures can significantly influence RBC adhesion because thermal stress alters both membrane structure and intracellular organization. Importantly, as has been shown for other heat-induced changes, these effects are time-dependent: short-term exposure produces mostly reversible changes, whereas prolonged or repeated heating progressively leads to a pro-adhesive and mechanically unstable RBC phenotype.

*Short-term febrile heating*: Short-term exposure to febrile-range temperatures (≈38–41 °C; seconds–minutes) increases lipid bilayer fluidity and enhances the lateral mobility of membrane proteins, thereby modifying the spatial distribution of adhesion-related membrane domains. At this stage, the RBC membrane remains structurally intact, and most adhesive changes are subtle and transient.

One important early effect of heating is the partial reduction in membrane surface charge [105]. Temperature-dependent rearrangement of membrane proteins [106] and local perturbations of glycocalyx organization may reduce electrostatic repulsion between RBCs and endothelial surfaces. Simultaneously, mild echinocytic transformation and transient changes in membrane curvature may locally expose membrane regions that are normally less accessible under physiological conditions [72].

Because PS externalization is a major pro-adhesive and pro-recognition signal, even small increases in externalized PS caused by heating may transiently enhance RBC interactions with endothelial cells, plasma proteins, macrophages, and components of the coagulation system [48,49].

Overall, short-term febrile heating produces a mildly pro-adhesive but largely reversible RBC state characterized by transient membrane remodeling, limited PS exposure, and subtle changes in cell–surface interactions.

*Long-term febrile heating*: Extended or repeated exposure to febrile-range temperatures causes RBCs to accumulate oxidative damage and structural membrane injuries. This process involves cytoskeletal remodeling, membrane vesiculation, and a slow loss of phospholipid asymmetry [62,107,108,109]. Studies have shown that temperature-dependent changes in membrane mechanics and cytoskeletal interactions occur in erythrocytes, suggesting that thermal stress can cause lasting alterations in membrane structure and flexibility [7,107]. Moreover, chronic stress exposure is associated with oxidative damage to membrane lipids and proteins, which can destabilize the cytoskeleton and impair cellular function [110].

Sustained heating disrupts the mechanical coupling between the lipid bilayer and spectrin-based membrane skeleton, promoting membrane protein clustering and irreversible reorganization of membrane domains [16,23,107,109]. In particular, thermal and oxidative stress may induce aggregation of band 3 complexes and destabilization of cytoskeletal anchoring structures, thereby creating localized adhesive hotspots on the RBC surface [33,85]. Simultaneously, progressive membrane vesiculation reduces membrane surface area and increases cell sphericity, impairing RBC deformability and prolonging cell–wall interactions within the microcirculation.

In addition, a sustained PS exposure strongly enhances RBC adhesion to endothelial cells, thrombospondin, fibrinogen, laminin, and activated macrophages, while simultaneously increasing procoagulant activity and RBC recognition by the reticuloendothelial system [38,111].

Oxidative stress becomes increasingly important during longer-term febrile heating [13,112,113]. The accumulation of reactive oxygen species promotes the oxidation of membrane lipids and proteins, weakening membrane integrity and altering surface receptor organization. Oxidative clustering of membrane proteins may further enhance abnormal RBC–RBC and RBC–endothelium interactions [23,114,115]. At the same time, increased intracellular viscosity and altered hemoglobin–membrane interactions elevate membrane tension and impair the dynamic membrane fluctuations that normally reduce the duration of adhesive contact [40,116,117,118].

Functionally, these cumulative effects may substantially impair microvascular flow. Pro-adhesive RBCs exhibit prolonged endothelial contact, impaired capillary transit, enhanced trapping within splenic slits, and increased participation in inflammatory and thrombotic processes. In low-shear regions of the microcirculation, enhanced adhesion may synergize with increased aggregation and reduced deformability to produce local flow disturbances and impaired oxygen delivery.

Importantly, endothelial cells themselves are temperature-sensitive [119,120,121]. Febrile-range temperatures can transiently influence endothelial activation state, local cytokine signaling, nitric oxide bioavailability, and expression of adhesion molecules [120,122]. Therefore, early RBC adhesion during fever likely reflects the combined thermal responses of circulating RBCs and the vascular endothelium rather than RBC alterations alone.

Overall, longer-term febrile heating promotes a progressively pro-adhesive RBC phenotype characterized by membrane destabilization, persistent PS exposure, oxidative remodeling, vesiculation, and impaired mechanical adaptability. These changes likely contribute to microcirculatory dysfunction, inflammation, thrombosis, and premature RBC clearance during severe or recurrent febrile states.

## 5. Clearance Signaling and Splenic Interaction

RBC clearance is governed by the integration of biochemical recognition signals and biomechanical filtration, primarily in the spleen. Under physiological conditions, RBCs repeatedly deform to pass through narrow splenic interendothelial slits, allowing the spleen to function as a mechanical quality-control system that selectively removes aged or damaged cells [123,124]. Febrile-range heating can influence both biochemical signaling and splenic filtration.

*Short-term febrile heating*: During short-term exposure to febrile temperatures, RBCs undergo early membrane and cytosolic alterations that may transiently increase clearance signaling without immediately causing irreversible damage. As intracellular Ca^2+^ rises, induced PS exposure on the outer membrane leaflet acts as an “eat-me” signal for macrophages, so that even transient exposure may increase the probability of splenic recognition.

At the same time, early oxidative and structural changes in the membrane may initiate limited band 3 clustering. Such clustering generates senescence-associated neoepitopes that bind naturally occurring anti-band 3 antibodies and complement components [25,125]. During short-term heating, these processes are generally modest and may remain partially reversible after restoration of normothermic conditions.

Mechanistically, short-term heating may initially boost RBC deformability by softening temperature-dependent membrane structures and decreasing intracellular resistance due to increased hemoglobin fluidity [107,126]. Moderate temperature increases can facilitate erythrocyte passage through narrow constrictions, suggesting that microcirculatory flow and splenic filtration may remain efficient during early thermal exposure [107]. Since splenic retention is heavily affected by decreased erythrocyte deformability and flexibility [127], short-term febrile heating probably does not cause immediate large-scale erythrocyte sequestration, despite early membrane signaling changes. Instead, short-term fever likely causes subtle, dynamic adjustments in erythrocyte clearance pathways, with significant splenic removal only occurring after more substantial structural or biochemical damage develops.

*Longer-term febrile heating*: With prolonged or repeated exposure, clearance-related alterations become progressively amplified and less reversible as erythrocytes accumulate structural and biochemical damage. Sustained cellular stress promotes persistent membrane scrambling and stabilization of PS exposure on the outer leaflet of the plasma membrane, a recognized hallmark of erythrocyte aging and eryptosis [44,108,128]. Surface-exposed PS functions as a potent “eat-me” signal that facilitates recognition by macrophages and cells of the reticuloendothelial system [129,130]. Consequently, stable PS exposure markedly enhances macrophage-mediated clearance and splenic sequestration of damaged erythrocytes, thereby accelerating their removal from the circulation [131,132].

Simultaneously, prolonged membrane remodeling and oxidative damage increase band 3 clustering and associated immune recognition pathways. Lutz et al. [125] and Pantaleo et al. [25] demonstrated that clustered band 3 serves as a major senescence signal that promotes the binding of naturally occurring antibodies and the deposition of complement. It should also be noted that band 3 clustering can cause PS to externalize on the RBC surface [53].

Mechanical retention in the spleen also becomes increasingly important during longer-term heat exposure. Progressive loss of membrane surface area, echinocyte-to-spherocyte transformation, dehydration, and cytoskeletal weakening reduce RBC deformability and impair transit through splenic interendothelial slits. Experimental and computational studies indicate that even moderate reductions in deformability can markedly increase splenic trapping [123,124]. In this context, febrile-range heating promotes a dual clearance mechanism: biochemical tagging through PS exposure and band 3 clustering, together with biomechanical retention due to impaired deformability.

Overall, longer-term febrile heating shifts RBCs toward a clearance-prone phenotype characterized by persistent membrane signaling, reduced mechanical resilience, vesiculation, and impaired splenic passage. These processes may contribute to shortened RBC lifespan, altered microcirculatory flow, and anemia during sustained fever and inflammatory states.

## 6. RBC Behavior Under Blood Flow: Long-Term Impact of Fever-Range Heating

Understanding how febrile-range heating affects RBCs involves combining multiscale modeling, microfluidic experiments, and research on mechanical fatigue and splenic filtration. Li et al. [15] describe the RBC membrane as a composite system in which the lipid bilayer and spectrin cytoskeleton contribute distinct yet interconnected mechanical properties. This complexity is especially relevant to longer-term febrile heating to febrile-range temperatures, as heat increases the bilayer-cytoskeleton attachment, leading to decreased deformability, slower recovery, and reduced red cell stability [5,6,15,69]. Because heat exposure induces irreversible changes in the mechanical properties of RBCs, it inevitably alters their flow behavior.

Matrai et al. [5] systematically studied how increased temperature affects RBC deformability and membrane stability across different species. They heated RBCs for 10 min before measuring deformability. Using ektacytometry, they found that heating to 40–43 °C significantly reduces RBC deformability, with the extent of impairment varying by species, sample composition, and heating protocol. Notably, responses in membrane stability were diverse and sometimes contradictory, suggesting that thermal effects on RBC mechanics are complex and context-dependent. These results emphasize that temperature influences micro-rheological behavior in a non-uniform way, shaped by both experimental conditions and biological differences.

Consistent with this approach, Quinn et al. [133] found that individual RBCs moving through narrow microchannels are highly sensitive to temperature changes, which they attributed to lower viscosities of both the membrane and the surrounding medium, leading to shorter relaxation times. Building on this, Rodríguez-Villarreal et al. [134] demonstrated that higher temperatures increase RBC lateral migration and flow behavior dependent on deformability, resulting in a significant expansion of the cell-free zone near the channel walls. Additionally, heated RBCs show increased transit times or retention during passage under confinement (via microchannels or slits), depending on the level of confinement and heating method [34]. These results suggest that heating not only alters intrinsic cell mechanics but also affects the distribution and margination of cells in the blood flow.

Furthermore, Liu et al. [34] demonstrated that thermal disruptions influence RBC biomechanics in various physiological contexts, including stretching, relaxation, capillary transit, and passage through splenic-like constrictions. These coordinated mechanical shifts have direct physiological effects. Since the spleen functions as a strict mechanical filter with specific thresholds for RBC deformability and shape [124], even slight temperature changes can increase the chance of RBC retention in the spleen.

Qiang et al. [77] emphasized the significance of loading history, revealing that cyclic mechanical stress speeds up RBC damage more than static deformation. During heat exposure, membranes become more flexible but less stable, potentially leading to cumulative injury.

*Interim conclusions*: Febrile-range heating has a complex, context-dependent effect on RBC behavior in flow. While higher temperatures can temporarily change deformability and cause redistribution in circulation, they also disrupt membrane mechanics, recovery, and stability. These effects influence not only the cells’ intrinsic properties but also their interactions with flow and confinement, leading to increased variation in transit times and a higher likelihood of retention in the spleen. Additionally, repeated mechanical stress under these conditions accelerates cumulative damage. Thus, febrile-range heating connects altered micro-rheology with reduced mechanical resilience, ultimately leading to RBC remodeling, retention, and clearance.

## 7. Intrinsic Heterogeneity of Circulating Red Blood Cells Determines Their Response to Febrile-Range Hyperthermia

The intrinsic heterogeneity of circulating RBCs likely plays a major role in determining how the RBC population responds to elevated temperatures, although this aspect remains insufficiently investigated [135]. Human blood is not a uniform population of cells but contains erythrocytes at different stages of their lifespan, exhibiting substantial variability in membrane composition, cytoskeletal organization, metabolic activity, antioxidant capacity, intracellular viscosity, deformability, and accumulated mechanical or oxidative damage [132,135,136,137,138]. As a consequence, individual RBCs are expected to differ considerably in their thermal tolerance and capacity to preserve membrane integrity during febrile-range heating.

The variability within RBC populations significantly influences how these cells adapt to temperature fluctuations, although this area needs more research. Blood contains diverse erythrocyte subgroups, including highly flexible discocytes, transitional forms, and less flexible echinocytes [139]. Each subtype responds differently to heat stress, so the overall impact of hyperthermia (up to 41 °C) likely reflects shifts in mechanical states rather than identical cellular responses.

At 37 °C, discocytes may temporarily soften due to increased lipid mobility, greater membrane fluctuations, and improved deformability. However, when the temperature nears 41 °C, RBCs that already have damage, an altered cytoskeleton, oxidative stress, or a smaller surface area become more prone to destabilization and often assume echinocytic shapes. Echinocytes exhibit reduced deformability due to loss of membrane area, dehydration, elevated cytosolic hemoglobin levels, and altered interactions between the membrane and the cytoskeleton. This makes them more vulnerable to mechanical trapping in the spleen and subsequent removal [139].

Temperature-induced rigid echinocytes cannot elongate under shear stress, causing blockages in small capillaries and increasing flow resistance. This results in localized tissue hypoxia and disrupted blood flow. The coexistence of flexible discocytes and rigid cells affects capillary congestion and accelerates splenic clearance at 37–41 °C [140,141].

## 8. Clinical and Pathophysiological Contexts

The impact of temperature on RBCs is particularly important in clinical settings with persistent or recurrent fever, systemic inflammation, oxidative stress, or cellular vulnerability from transfusions. In these cases, a febrile-range temperature not only acts as a stand-alone stressor but also exacerbates existing RBC dysfunction.

*Infections and malaria*: Malaria provides one of the most prominent examples in which fever, RBC mechanical impairment, and microvascular obstruction converge. In *Plasmodium falciparum* infection, both infected and uninfected RBCs show reduced deformability, which contributes to impaired microcirculatory flow, splenic retention, and severe disease outcomes [142,143]. Experimental studies have shown that febrile-range temperature can further stiffen parasitized RBCs and exacerbate mechanical abnormalities, potentially increasing vascular obstruction and splenic clearance [2,144]. Thus, fever may intensify malaria pathophysiology by worsening RBC mechanical dysfunction.

*Inflammation and sepsis*: In sepsis and systemic inflammation, RBCs are exposed simultaneously to fever, oxidative stress, inflammatory mediators, metabolic disturbances, and altered plasma composition. These factors promote changes in RBC morphology, deformability, membrane stability, and oxygen-handling capacity [145]. Oxidative stress and Ca^2+^ entry can induce eryptosis, characterized by cell shrinkage, membrane blebbing, and phosphatidylserine exposure, thereby accelerating RBC clearance [38,146]. Febrile-range heating may therefore amplify inflammation-induced membrane remodeling and clearance signaling.

*Transfusion medicine*: Stored RBCs are already mechanically and biochemically compromised due to the storage lesion, which includes ATP depletion, oxidative damage, vesiculation, membrane surface loss, altered ion homeostasis, and progressive loss of deformability [147,148]. These changes may reduce the capacity of stored RBCs to withstand additional thermal stress after transfusion. Long-stored cells are more likely to show non-discocytic morphology, increased osmotic fragility, and impaired deformability, making them potentially more susceptible to febrile or inflammatory environments [147,149,150]. Thus, fever may further accelerate functional decline or premature clearance of transfused RBCs.

*Neonatal and critical care*: RBC temperature sensitivity may be especially relevant in vulnerable populations, including premature infants and critically ill patients. Preterm neonates frequently receive adult donor RBC transfusions, despite important physiological differences between fetal/neonatal and adult erythrocytes, including hemoglobin composition, metabolism, oxidative stress handling, and membrane properties [151,152,153]. In critical care, anemia, inflammation, transfusion, and fever often coexist, creating conditions in which RBC deformability and survival may be compromised. These considerations suggest that febrile stress could have disproportionate effects in patients with limited microvascular reserve or reduced antioxidant capacity.

Overall, febrile-range heating should be viewed as a clinically relevant modifier of RBC function. Its impact is likely greatest when combined with infection, inflammation, storage lesions, or functional vulnerability, in which even modest changes in deformability, aggregation, clearance signaling, or hemolytic susceptibility may affect microvascular perfusion and oxygen delivery.

## 9. Protective Mechanisms Limiting Heat-Induced RBC Damage

Despite the changes caused by febrile-range hyperthermia in structure and biochemistry, erythrocytes have innate mechanisms that delay irreversible membrane damage and maintain their mechanical function under thermal stress. Since mature RBCs do not have nuclei or organelles, these defenses rely solely on existing metabolic pathways, antioxidant systems, membrane remodeling, and protective factors from plasma, rather than new protein production [16,111,154].

The antioxidant defense system is the main mechanism that limits heat-induced damage. Higher temperatures speed up hemoglobin auto-oxidation, leading to more reactive oxygen species (ROS). These ROS damage membrane lipids and cytoskeletal proteins, cause band 3 clustering, and promote membrane-bound hemoglobin formation [61,155,156]. Reduced glutathione (GSH), along with enzymes like glutathione peroxidase, glutathione reductase, catalase, superoxide dismutase, and the pentose phosphate pathway, reduces oxidative damage and helps maintain membrane integrity [155,156]. However, as RBC age, these defenses weaken, increasing their vulnerability to extended thermal stress.

A second protective mechanism is physiological membrane vesiculation. Rather than being a degenerative process, this controlled shedding of vesicles intentionally removes oxidized Hb, damaged membrane proteins, and components linked to cell aging. This process helps delay hemolysis and preserves membrane stability, even as the membrane gradually deteriorates [60,62].

Maintaining membrane lipid asymmetry is essential for RBC survival. ATP-dependent aminophospholipid translocases regularly move PS back to the inner membrane layer, avoiding early recognition by macrophages, preventing endothelial adhesion, and activating coagulation pathways [56,157,158]. Longer-term febrile heating, oxidative stress, ATP depletion, and increased intracellular Ca^2+^ gradually weaken these transport mechanisms, making eryptosis and splenic clearance more likely [56,158].

The spectrin–actin cytoskeleton displays notable structural resilience. Temporary weakening of bilayer–cytoskeleton interactions at febrile temperatures can temporarily increase membrane flexibility and help capillaries, while permanent cytoskeletal damage usually occurs from longer-term febrile heating combined with oxidative stress and calcium overload [4,5,7]. Additionally, good nutritional status enhances RBC resistance to heat and oxidative damage. Nutrients such as vitamins C and E, selenium-dependent glutathione peroxidase, zinc-containing antioxidant enzymes, and other micronutrients help maintain membrane integrity by reducing oxidative harm [159,160].

Recent research indicates that diets rich in antioxidants and anti-inflammatory substances improve RBC membrane composition, cytoskeletal stability, and flow properties, potentially boosting resilience to environmental challenges [159,161,162,163]. While direct evidence linking nutritional strategies to better thermal tolerance is limited, supporting optimal antioxidant function may help prevent the shift from reversible membrane softening to irreversible heat-induced membrane changes.

## 10. Conclusions

A febrile-range temperature induces a coordinated, multiscale response in red blood cells, involving the membrane, cytoskeleton, cytosol, and cell–cell interactions. Although moderate heating may transiently increase RBC deformability and improve flow adaptability, this apparent mechanical benefit reflects a shift toward a more compliant but mechanically vulnerable state. Elevated temperature alters lipid bilayer organization, weakens membrane–cytoskeleton coupling, modifies hemoglobin conformation and intracellular viscosity, perturbs ion homeostasis, and promotes vesiculation, PS exposure, and protein clustering. Together, these processes progressively reduce the mechanical safety margin of RBCs.

Importantly, the effects of heating are strongly time-dependent. Short-term exposure primarily induces reversible physicochemical adaptations, including membrane softening, a transient increase in deformability, and moderate vesiculation. In contrast, prolonged or repeated heating drives cumulative structural remodeling characterized by cytoskeletal weakening, dehydration, altered aggregation behavior, increased fragility, and activation of clearance pathways. Eventually, these changes irreversibly impair RBCs’ ability to withstand repeated mechanical stress during circulation and splenic filtration.

The reviewed evidence further demonstrates that temperature-dependent RBC remodeling cannot be interpreted solely as a membrane phenomenon. Instead, RBC mechanics emerge from tightly coupled interactions between membrane organization, intracellular hemoglobin dynamics, water structuring, ion transport, and external hemodynamic forces. This integrated perspective helps explain why fever can substantially influence RBC function even in the absence of overt hemolysis.

Clinically, fever-associated RBC remodeling is likely to be particularly important in conditions involving infection, inflammation, oxidative stress, blood transfusion, or pre-existing microvascular dysfunction. In such settings, thermally induced changes in deformability, aggregation, fragility, and clearance signaling may contribute to impaired microcirculatory perfusion and altered oxygen delivery. Overall, febrile-range heating should be viewed not merely as a passive environmental factor but as an active modulator of RBC mechanical and physiological behavior.

Table 2 summarizes the main differences between the effects of short-term and longer-term febrile heating on key RBC properties.

## 11. Limitations

When interpreting the findings in this review, several limitations must be considered. The existing literature is highly diverse in terms of experimental design, such as variations in temperature range, exposure duration, sample preparation, measurement methods, and examined physiological endpoints. These methodological differences make direct comparisons difficult and prevent a quantitative synthesis.

Secondly, few studies have systematically explored how thermal effects change over time under febrile conditions relevant to physiology. Most research focuses on a single exposure duration or temperature, making it challenging to clearly distinguish shifts from reversible adaptive responses to permanent membrane remodeling.

Third, although this review mainly discusses healthy human RBCs, some mechanistic ideas are backed by evidence from related experimental systems or pathological states. While these studies offer useful mechanistic insights, their direct relevance to healthy RBCs during physiological fever should be considered with caution.

Another key limitation is the inherent heterogeneity within circulating RBC populations. These cells vary significantly in age, membrane composition, cytoskeletal structure, metabolic function, antioxidant levels, intracellular viscosity, deformability, and accumulated mechanical or oxidative damage. As a result, the population-averaged responses discussed in this review probably conceal substantial variability in thermal sensitivity among individual cells.

Most of the available evidence has been gathered from in vitro studies conducted under controlled laboratory conditions. In living organisms, however, fever is accompanied by inflammation, changes in plasma composition, oxidative stress, cytokine release, and blood flow alterations, all of which can significantly influence erythrocyte responses to increased temperature.

## 12. Future Perspectives

Future research should aim to establish a more integrated and physiologically relevant understanding of RBC responses to febrile-range temperature. A major priority is to integrate membrane biophysics, intracellular dynamics, rheology, and microcirculatory modeling into unified multiscale frameworks that describe how thermal stress propagates from molecular changes to alterations in systemic flow.

Particular attention must be paid to the coupling between membrane organization and intracellular hemoglobin dynamics. Emerging evidence suggests that temperature-dependent changes in hemoglobin conformation, hydration, and cytosolic viscosity strongly influence membrane mechanics and force transmission. Advanced imaging, spectroscopy, and computational modeling approaches may help clarify these interactions and identify new biomarkers of thermally induced RBC dysfunction.

Future studies should also distinguish more clearly between short-term adaptive responses and progressive mechanical fatigue. Longitudinal experiments involving repeated febrile cycles, physiological flow conditions, and splenic-mimetic filtration systems may provide important insights into cumulative RBC damage and lifespan regulation.

A promising research avenue is examining heat sensitivity in key RBC groups, including stored units, neonatal erythrocytes, sickle cells, diabetic RBCs, and cells from septic patients. These studies will enhance our understanding of why specific patient groups are especially prone to microcirculatory issues during fever and inflammation.

From a translational perspective, temperature-dependent RBC mechanics may have implications for transfusion medicine, infectious disease management, intensive care, and microvascular diagnostics. Quantitative assessment of RBC thermal sensitivity could potentially serve as a marker of cellular resilience, storage quality, or severity of a clinical condition. Furthermore, integrating rheological, dielectric, and imaging approaches may enable the development of novel diagnostic tools to monitor the functional state of RBCs under thermal or inflammatory stress.

Overall, future work will have to move beyond viewing fever merely as a thermal perturbation and instead to consider it as a dynamic biomechanical and biochemical modulator of RBC physiology.

## Figures and Tables

**Table 1:** Representative experimental studies investigating the effects of temperature on RBCs.

Study	Experimental Model	Temperature (°C)	Exposure Duration	Method	Principal Finding
Waugh & Evans [7]	Human RBCs (in vitro)	2–50	Minutes	Micropipette aspiration	Membrane shear modulus decreases with increasing temperature
Park et al. [8]	Human RBCs (in vitro)	25–41	Minutes	Diffraction phase microscopy	Increased membrane fluctuations at elevated temperature
Nash & Meiselman [6]	Human RBCs (in vitro)	37–50	≥30 min	Ektacytometry/filtration	Reduced deformability after prolonged heating
Kozlova et al. [4]	Human RBCs (in vitro)	37–43	30 min	Atomic force microscopy (AFM)	Spectrin network remodeling and nanodefect formation
Sheikhhassani et al. [9]	Human RBCs (in vitro)	40	Minutes	Optical tweezers	Temperature-dependent reduction in RBC stiffness
Matrai et al. [5]	Human RBCs (in vitro)	40–43	1 h	Ektacytometry and osmotic fragility assays	Reduced deformability and membrane stability
Vodyanoy et al. [10]	Human RBCs (in vitro)	37–40	Minutes	Light microscopy/vesicle analysis	Approximately threefold increase in vesicle release
Moore et al. [11]	Fresh human whole blood	37–40	Minutes	High-resolution live-cell light microscopy	Echinocytosis associated with increased vesiculation

**Table 2:** Summary of the time-dependent effects of febrile-range hyperthermia on major red blood cell properties.

RBC Property	Short-Term Heating (s–min)	Prolonged Heating (≥30 min)	Principal Mechanism (s)	Relative Section
Membrane lipid asymmetry	Generally preserved	Progressive phosphatidylserine externalization	ATP depletion, Ca^2+^ influx, scramblase activation, reduced flippase activity	Section 2.5
Membrane mechanics	Increased membrane fluidity; reduced shear modulus; increased membrane fluctuations; reversible membrane softening	Progressive membrane destabilization; cytoskeletal disruption; reduced mechanical stability	Lipid fluidization followed by oxidative remodeling and spectrin disruption	Section 2.6
Vesiculation	Slight increase in vesicle release	Markedly increased vesiculation	Oxidative stress, membrane budding, echinocyte formation	Section 3.1
Cell morphology	Predominantly discocytes; occasional reversible echinocytes	Discocyte-to-echinocyte transition; irreversible shape abnormalities	Cytoskeletal remodeling, membrane loss, altered bilayer–cytoskeleton interactions	Section 3.2
Deformability	Transient increase in deformability	Progressive decline in deformability	Initial membrane softening followed by oxidative damage, membrane-bound hemoglobin accumulation, and dehydration	Section 4.1
Membrane fragility	Little or no significant change	Increased osmotic and mechanical fragility	Oxidative injury, membrane protein modification, membrane loss	Section 4.2
Aggregation	Minor reduction or no appreciable change	Altered aggregation depending on membrane remodeling and plasma environment	Changes in membrane flexibility, surface properties, and cell morphology	Section 4.3
Endothelial adhesion	Minimal effect in healthy RBCs	Increased endothelial adhesion	Phosphatidylserine exposure, oxidative stress, altered membrane proteins	Section 4.4
Clearance signals	Minimal activation	Increased band 3 clustering, phosphatidylserine exposure, enhanced macrophage recognition	Oxidative damage, eryptosis, membrane remodeling	Section 5

## Data Availability

No new data were created or analyzed in this study. Data sharing is not applicable to this article.

## Abbreviations

The following abbreviations are used in this manuscript:

RBC	Red blood cell
Hb	Hemoglobin
MBHb	Membrane-bound hemoglobin
ATP	Adenosine triphosphate
PS	Phosphatidylserine
AFM	Atomic force microscopy
MCHC	Mean corpuscular hemoglobin concentration
